# Impact of the double expression of MYC and BCL2 on outcomes of localized primary gastric diffuse large B-cell lymphoma patients in the rituximab era

**DOI:** 10.1038/bcj.2016.88

**Published:** 2016-09-30

**Authors:** A Kawajiri, D Maruyama, A M Maeshima, J Nomoto, S Makita, H Kitahara, K-i Miyamoto, S Fukuhara, T Suzuki, W Munakata, K Tajima, J Itami, H Taniguchi, Y Kobayashi, K Tobinai

**Affiliations:** 1 Department of Hematology, National Cancer Center Hospital, Tokyo, Japan; 2 Department of Pathology and Clinical Laboratory, National Cancer Center Hospital, Tokyo, Japan; 3 Department of Radiation Oncology, National Cancer Center Hospital, Tokyo, Japan

Diffuse large B-cell lymphoma (DLBCL) is a common subtype of primary gastric lymphoma, accounting for 50–60% of cases.^1^ Prior to the rituximab era, a Japanese phase II trial evaluated three courses of cyclophosphamide, doxorubicin, vincristine and prednisolone (CHOP), followed by 40.5 Gy of involved field radiotherapy (IFRT) for localized (stage I, II_1_ based on the Lugano Staging System for Gastrointestinal Lymphoma^2^) primary gastric DLBCL (PG-DLBCL) and the study yielded good therapeutic results.^3^ Rituximab plus CHOP (R-CHOP) therapy has been shown to improve the prognosis of DLBCL patients over that of patients treated with CHOP.^4, 5^ The efficacy of three cycles of R-CHOP followed by IFRT for localized DLBCL was evaluated in a phase II trial by the Southwest Oncology Group and also showed favorable results.^6^ On the basis of these findings, a rituximab-containing regimen, particularly three cycles of R-CHOP followed by IFRT, is regarded as one of the standard therapies for localized DLBCL including PG-DLBCL. Recent studies demonstrated that the double rearrangement (double hit) of the *MYC* and *BCL2* genes and the double expression of the MYC and BCL2 proteins were associated with a poor prognosis for patients with nodal DLBCL.^7, 8, 9, 10^ However, the clinical impact of the double expression of MYC and BCL2 on PG-DLBCL remains unknown. We retrospectively analyzed patients with localized PG-DLBCL who were initially treated with a rituximab-containing regimen, with a focus on the status of MYC and BCL2.

We retrospectively analyzed 52 consecutive patients newly diagnosed with localized PG-DLBCL at our institution between 2003 and 2013. They were initially treated or planned to be treated with a rituximab-containing regimen. All patients underwent standard staging procedures, including upper gastrointestinal endoscopy, bone marrow aspiration or biopsy, computed tomography, and/or fluorine-18-2-fluoro-2-deoxy-D-glucose positron emission tomography/computed tomography and were assigned a clinical stage according to Lugano Staging System for Gastrointestinal Lymphomas.^2^ This retrospective study was approved by the Institutional Review Board of the National Cancer Center in Japan. It was conducted in accordance with the international ethical recommendations stated in the Declaration of Helsinki and Japanese Good Clinical Practice Guidelines. Histopathological diagnoses were made and reviewed by two experienced hematopathologists (AMM and HT) according to the criteria of the WHO classification.^11^ An immunohistochemical analysis of formalin-fixed, paraffin-embedded tissues was performed using a panel of monoclonal antibodies. MYC immunoreactivity was considered positive when the MYC protein was expressed in more than 40% of tumor cells. BCL2 was considered positive when the BCL2 protein was expressed in more than 70% of the tumor cells.^7^ Lymphoma cells were assigned a germinal center B-cell-like (GCB) or non-GCB phenotype using the Hans algorithm for cell-of-origin subtyping.^12^ A fluorescence *in situ* hybridization analysis was performed on formalin-fixed, paraffin-embedded tissue sections. The following probes were used: an LSI *MYC* dual-color break apart rearrangement probe and LSI *IGH/BCL2* dual-color dual-fusion translocation probe (Vysis, Downers Grove, IL, USA). Overall survival (OS) was defined as the interval between the date of diagnosis and date of death or last follow-up. Progression-free survival was defined as the interval between the date of diagnosis and date of death, disease progression or last follow-up. Survival analyses were performed using Kaplan–Meier's method and compared using the log-rank test. All *P*-values were based on two-sided tests and *P*-values <0.05 were considered significant. Statistical analyses were performed using EZR version 1.27.^13^

The characteristics of all 52 patients are shown in Table 1. Twenty-four patients (46%) were male and 28 (54%) were female with a median age of 62 years (range: 29–85). Performance statuses were 0–1 in 47 patients (90%). Thirty patients (58%) presented with stage I disease, 15 (29%) with stage II_1_, 2 (4%) with stage II_2_ and 5 (9%) with stage IIE. Most patients (47 patients: 90%) had a low or low-intermediate risk according to the International Prognostic Index.^14^ Serum lactate dehydrogenase levels were normal in 45 patients (87%). Forty-eight patients (92%) lacked B symptoms.

The details of treatments were as follows: 43 patients (83%) received R-CHOP followed by IFRT, 7 (13%) received R-CHOP alone, 1 (2%) underwent total gastrectomy followed by rituximab because the patient denied chemotherapy and IFRT, and 1 (2%) received CHOP plus IFRT. The median number of CHOP cycles was three (range: 2–8). Most patients (43 patients: 83%) were treated with R-CHOP plus IFRT. The median dose of IFRT was 40 Gy (range: 30–40 Gy).

The Ki-67 index was ⩾90% in 25 out of 46 evaluable patients (54%). The cell-of-origin subtype was assigned in 48 of the 52 patients (30 patients (63%) GCB and 18 patients (37%) non-GCB). The double expression of MYC and BCL2 was assessed in 47 out of 52 patients (90%), and confirmed in 7 (15%). *MYC* break apart was detected in 1 out of 24 patients (4%) evaluated by fluorescence *in situ* hybridization. *IGH/BCL2* fusion was confirmed in 1 out of 27 evaluable patients (4%). *MYC* break apart and *IGH/BCL2* fusion were both assessed in 22 patients including 5 with the double expression of MYC and BCL2. However, no patient had the double hit of *MYC* break apart and *IGH/BCL2* fusion.

The median follow-up duration was 76 months (range: 4–127 months). Fifty patients (96%) achieved complete response, and the remaining two patients without the double expression of MYC and BCL2 had primary refractory disease. The estimated 5-year OS and progression-free survival rates of all 52 patients were 90% (95% confidence interval (CI), 75–96%) and 89% (95% CI, 75–95%), respectively (Figure 1a). The estimated 5-year OS rates of patients with and without the double expression of MYC and BCL2 were 100% and 87% (95% CI, 69–95%), respectively (Figure 1b). No significant difference was observed between the two cohorts (*P*=0.74). The estimated 5-year OS rates of the GCB phenotype and non-GCB phenotype were 86% (95% CI, 63–96%) and 93% (95% CI, 59–99%), respectively, and this difference was not significant (*P*=0.99). Six out of 52 patients died. The causes of death were as follows; two were due to the progression of DLBCL without the double expression of MYC and BCL2, and the remaining four died of other cancers without DLBCL.

The results of the present study revealed the good prognosis of patients with localized PG-DLBCL treated with rituximab-containing chemotherapy with or without IFRT, and demonstrated that double expression of MYC and BCL2 did not influence patient outcomes. Although CHOP followed by IFRT is regarded as one of the standard therapies for localized PG-DLBCL, information in the rituximab era has been limited. Our study confirmed the efficacy and feasibility of R-CHOP plus IFRT, and this strategy is considered to be a reasonable standard therapy for localized PG-DLBCL in the rituximab era.

The prognosis of nodal DLBCL patients with the double expression of MYC and BCL2 treated with R-CHOP therapy was significantly worse in previous studies,^7, 8, 9, 10^ and it was also shown that the prognosis of patients with the double expression and double hit was not significantly different.^7^ There is currently no standard therapy for DLBCL patients with the double hit or double expression. Several studies have suggested that more intensive chemotherapies than R-CHOP or stem cell transplantation may overcome the poor prognosis of double hit lymphoma.^15^ However, these hypotheses have mainly been discussed for nodal DLBCL. The results of the present study suggest that R-CHOP followed by IFRT is sufficient for most localized PG-DLBCL patients with the double expression of MYC and BCL2. On the other hand, as no double hit patients were included, it was not possible to evaluate the impact of the translocation in our study.

There were several limitations to this study. This was a retrospective analysis conducted at a single institution. The number of patients and events was too small to draw any definitive conclusions. Although most patients received R-CHOP plus IFRT, 17% were treated with other regimens. Specimen availability differed in each patient; therefore, the profiles of all patients were not assessed for the double hit or double expression of MYC and BCL2.

In conclusion, the results of the present study showed good prognoses, and suggest that the double expression of MYC and BCL2 did not influence the outcomes of localized PG-DLBCL patients treated with rituximab-containing chemotherapy with or without RT. Further investigations are needed in order to confirm our results.

## Figures and Tables

**Figure 1:**
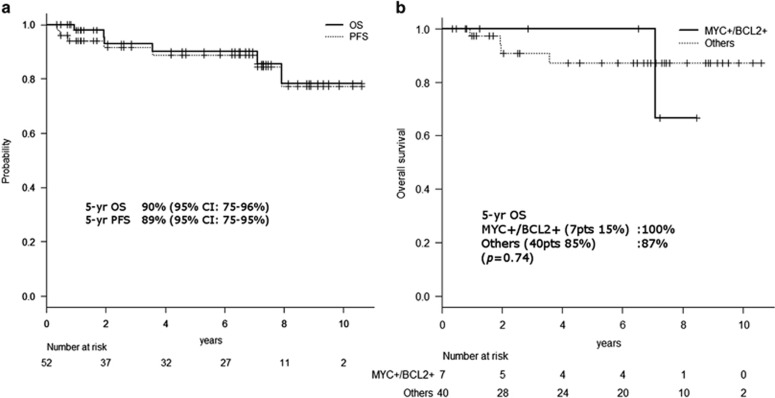
(**a**) Overall and progression-free survival (PFS) rates of all 52 patients. The estimated 5-year OS and PFS rates of all 52 patients were 90% (95% CI, 75–96%) and 89% (95% CI, 75–95%), respectively. (**b**) Overall survival rates according to the MYC/BCL2 status. Kaplan–Meier curves of OS rates according to the MYC/BCL2 status. No significant difference was observed between the two groups.

**Table 1:** Patient characteristics

N=*52*	n	*(%)*
Median age (range)	62 years	(29–85)
Male/female	24/28	(46/54)
PS 0–1	47	(90)
		
*Stage*^a^		
Stage I	30	(58)
Stage II_1_	15	(29)
Stage II_2_	2	(4)
Stage IIE	5	(9)
Normal serum LDH	45	(87)
IPI low, low–intermediate	47	(90)
B symptoms absent	48	(92)

Abbreviations: IPI, International Prognostic Index; LDH, lactate dehydrogenase; PS, performance status.

a Lugano Staging System for gastrointestinal lymphomas.

## References

[bib1] Koch P, Probst A, Berdel WE, Willich NA, Reinartz G, Brockmann J et al. Treatment results in localized primary gastric lymphoma: data of patients registered within the German multicenter study (GIT NHL 02/96). J Clin Oncol 2005; 23: 7050–7059. 16129843 10.1200/JCO.2005.04.031

[bib2] Rohatiner A, d'Amore F, Coiffier B, Crowther D, Gospodarowicz M, Isaacson P et al. Report on a workshop convened to discuss the pathological and staging classifications of gastrointestinal tract lymphoma. Ann Oncol 1994; 5: 397–400. 8075046 10.1093/oxfordjournals.annonc.a058869

[bib3] Ishikura S, Tobinai K, Ohtsu A, Nakamura S, Yoshino T, Oda I et al. Japanese multicenter phase II study of CHOP followed by radiotherapy in stage I-II, diffuse large B-cell lymphoma of the stomach. Cancer Sci 2005; 96: 349–352. 15958057 10.1111/j.1349-7006.2005.00051.x PMC11159192

[bib4] Coiffier B, Lepage E, Briere J, Herbrecht R, Tilly H, Bouabdallah R et al. CHOP chemotherapy plus rituximab compared with CHOP alone in elderly patients with diffuse large-B-cell lymphoma. N Engl J Med 2002; 346: 235–242. 11807147 10.1056/NEJMoa011795

[bib5] Pfreundschuh M, Trümper L, Österborg A, Pettengell R, Trneny M, Imrie K et al. CHOP-like chemotherapy plus rituximab versus CHOP-like chemotherapy alone in young patients with good-prognosis diffuse large-B-cell lymphoma: a randomised controlled trial by the MabThera International Trial (MInT) Group. Lancet Oncol 2006; 7: 379–391. 16648042 10.1016/S1470-2045(06)70664-7

[bib6] Persky DO, Unger JM, Spier CM, Stea B, LeBlanc M, McCarty MJ et al. Phase II study of rituximab plus three cycles of CHOP and involved-field radiotherapy for patients with limited-stage aggressive B-cell lymphoma: Southwest Oncology Group study 0014. J Clin Oncol 2008; 26: 2258–2263. 18413640 10.1200/JCO.2007.13.6929

[bib7] Green TM, Young KH, Visco C, Xu-Monette ZY, Orazi A, Go RS et al. Immunohistochemical double-hit score is a strong predictor of outcome in patients with diffuse large B-cell lymphoma treated with rituximab plus cyclophosphamide, doxorubicin, vincristine, and prednisone. J Clin Oncol 2012; 30: 3460–3467. 22665537 10.1200/JCO.2011.41.4342

[bib8] Johnson NA, Slack GW, Savage KJ, Connors JM, Ben-Neriah S, Rogic S et al. Concurrent expression of MYC and BCL2 in diffuse large B-cell lymphoma treated with rituximab plus cyclophosphamide, doxorubicin, vincristine, and prednisone. J Clin Oncol 2012; 30: 3452–3459. 22851565 10.1200/JCO.2011.41.0985 PMC3454768

[bib9] Perry AM, Alvarado-Bernal Y, Laurini JA, Smith LM, Slack GW, Tan KL et al. MYC and BCL2 protein expression predicts survival in patients with diffuse large B-cell lymphoma treated with rituximab. Br J Haematol 2014; 165: 382–391. 24506200 10.1111/bjh.12763

[bib10] Hu S, Xu-Monette ZY, Tzankov A, Green T, Wu L, Balasubramanyam A et al. MYC/BCL2 protein coexpression contributes to the inferior survival of activated B-cell subtype of diffuse large B-cell lymphoma and demonstrates high-risk gene expression signatures: a report from The International DLBCL Rituximab-CHOP Consortium Program. Blood 2013; 121: 4021–4031, quiz 250. 23449635 10.1182/blood-2012-10-460063 PMC3709650

[bib11] Swerdlow SH, Campo E, Harris NL, Jaffe ES, Pileri SA, Stein H et al. WHO Classification of Tumours of Haematopoietic and Lymphoid Tissues. International Agency for Research on Cancer (IARC): Lyon, France, 2008.

[bib12] Hans CP, Weisenburger DD, Greiner TC, Gascoyne RD, Delabie J, Ott G et al. Confirmation of the molecular classification of diffuse large B-cell lymphoma by immunohistochemistry using a tissue microarray. Blood 2004; 103: 275–282. 14504078 10.1182/blood-2003-05-1545

[bib13] Kanda Y. Investigation of the freely available easy-to-use software 'EZR' for medical statistics. Bone Marrow Transplant 2013; 48: 452–458. 23208313 10.1038/bmt.2012.244 PMC3590441

[bib14] A predictive model for aggressive non-Hodgkin's lymphoma. The International Non-Hodgkin's Lymphoma Prognostic Factors Project. N Engl J Med 1993; 329: 987–994. 8141877 10.1056/NEJM199309303291402

[bib15] Petrich AM, Gandhi M, Jovanovic B, Castillo JJ, Rajguru S, Yang DT et al. Impact of induction regimen and stem cell transplantation on outcomes in double-hit lymphoma: a multicenter retrospective analysis. Blood 2014; 124: 2354–2361. 25161267 10.1182/blood-2014-05-578963

